# Assessment of spongy moth infestation impacts on forest productivity and carbon loss using the Sentinel-2 satellite remote sensing and eddy covariance flux data

**DOI:** 10.1186/s13717-024-00520-w

**Published:** 2024-05-14

**Authors:** Nur Hussain, Alemu Gonsamo, Shusen Wang, M. Altaf Arain

**Affiliations:** 1 School of Earth, Environment and Society and McMaster Centre for Climate Change, McMaster University, Hamilton, ON L8S 4K1 Canada; 2 Canada Centre for Remote Sensing, Natural Resources Canada, 1280 Main Street West, Ottawa, ON Canada

**Keywords:** Spongy moth, Infestation, Carbon, Gross ecosystem productivity, Leaf area index, Temperate forest, Remote sensing, Sentinel-2, Eddy covariance

## Abstract

**Background:**

Deciduous forests in eastern North America experienced a widespread and intense spongy moth (*Lymantria dispar*) infestation in 2021. This study quantified the impact of this spongy moth infestation on carbon (C) cycle in forests across the Great Lakes region in Canada, utilizing high-resolution (10 × 10 m^2^) Sentinel-2 satellite remote sensing images and eddy covariance (EC) flux data. Study results showed a significant reduction in leaf area index (LAI) and gross primary productivity (GPP) values in deciduous and mixed forests in the region in 2021.

**Results:**

Remote sensing derived, growing season mean LAI values of deciduous (mixed) forests were 3.66 (3.18), 2.74 (2.64), and 3.53 (2.94) m^2^ m^−2^ in 2020, 2021 and 2022, respectively, indicating about 24 (14)% reduction in LAI, as compared to pre- and post-infestation years. Similarly, growing season GPP values in deciduous (mixed) forests were 1338 (1208), 868 (932), and 1367 (1175) g C m^−2^, respectively in 2020, 2021 and 2022, showing about 35 (22)% reduction in GPP in 2021 as compared to pre- and post-infestation years. This infestation induced reduction in GPP of deciduous and mixed forests, when upscaled to whole study area (178,000 km^2^), resulted in 21.1 (21.4) Mt of C loss as compared to 2020 (2022), respectively. It shows the large scale of C losses caused by this infestation in Canadian Great Lakes region.

**Conclusions:**

The methods developed in this study offer valuable tools to assess and quantify natural disturbance impacts on the regional C balance of forest ecosystems by integrating field observations, high-resolution remote sensing data and models. Study results will also help in developing sustainable forest management practices to achieve net-zero C emission goals through nature-based climate change solutions.

## Introduction

Forest ecosystems cover more than 30% of the terrestrial area and play a crucial role in the global carbon (C) cycle through the processes of photosynthesis and respiration (FAO 2010; Ahmed 2018). The balance between these two opposing fluxes determines whether the forest ecosystem is C sink or source (DeLucia et al. 2007; Litton et al. 2007; Schmid et al. 2016; Chi et al. 2021). Forests have consistently demonstrated higher levels of gross primary productivity (GPP) and established the Earth’s most substantial C pools (Peters et al. 2007). Forests in North America are estimated to contribute approximately 76% of the region’s net terrestrial C sequestration (Zhao et al. 2021). In Canada, forest ecosystems have accumulated on average 173 million tons of C per year over much of the past century (Gray et al. 2006; Hengeveld et al. 2008). However, this rate of C sequestration can be influenced by natural disturbances such as wildfires and insect infestations (Kurz et al. 2002; Kalamandeen et al. 2023).

In North America, frequent outbreaks of insect infestations including mountain pine beetle (*Dendroctonus ponderosae*) infestation in western parts and spongy moth (*Lymantria dispar*) infestations in eastern regions have been the major factors impacting forest growth, health and C balance (Kurz et al. 2002). The spongy moth is a non-native species originally from Europe and Asia (Joria et al. 1991; Wang et al. 2022), that was first accidentally introduced in Boston area in USA in 1869 (Williams et al. 1985; Picq et al. 2023). Since then, it has expanded its range from New England to southward in Virginia to North Carolina and westward in Wisconsin, Michigan and the Great Lakes regions in USA and Canada (De Beurs and Townsend 2008; Hajek et al. 2021). Spongy month causes defoliation of various deciduous and mixed forests, including oak (*Quercus*), birch (*Betula*), aspen (*Populus*), sugar maple (*Acer saccharum*), American beech (*Fagus grandifolia*), balsam fir (*Abies balsamea*), and in sever infestation cases eastern white pine (*Pinus strobus*) and Colorado blue spruce (*Picea pungens*). The spongy moth’s life cycle involves egg dispersion before April, with early-stage caterpillars persisting until mid-May, late-stage caterpillars emerging in June, pupae developing in mid-July, and adult moths appearing by mid-August (Government of Ontario 2024). Defoliation typically begins in the early caterpillar stage and intensifies throughout the late caterpillar stage from June to August.

**Fig. 1:**
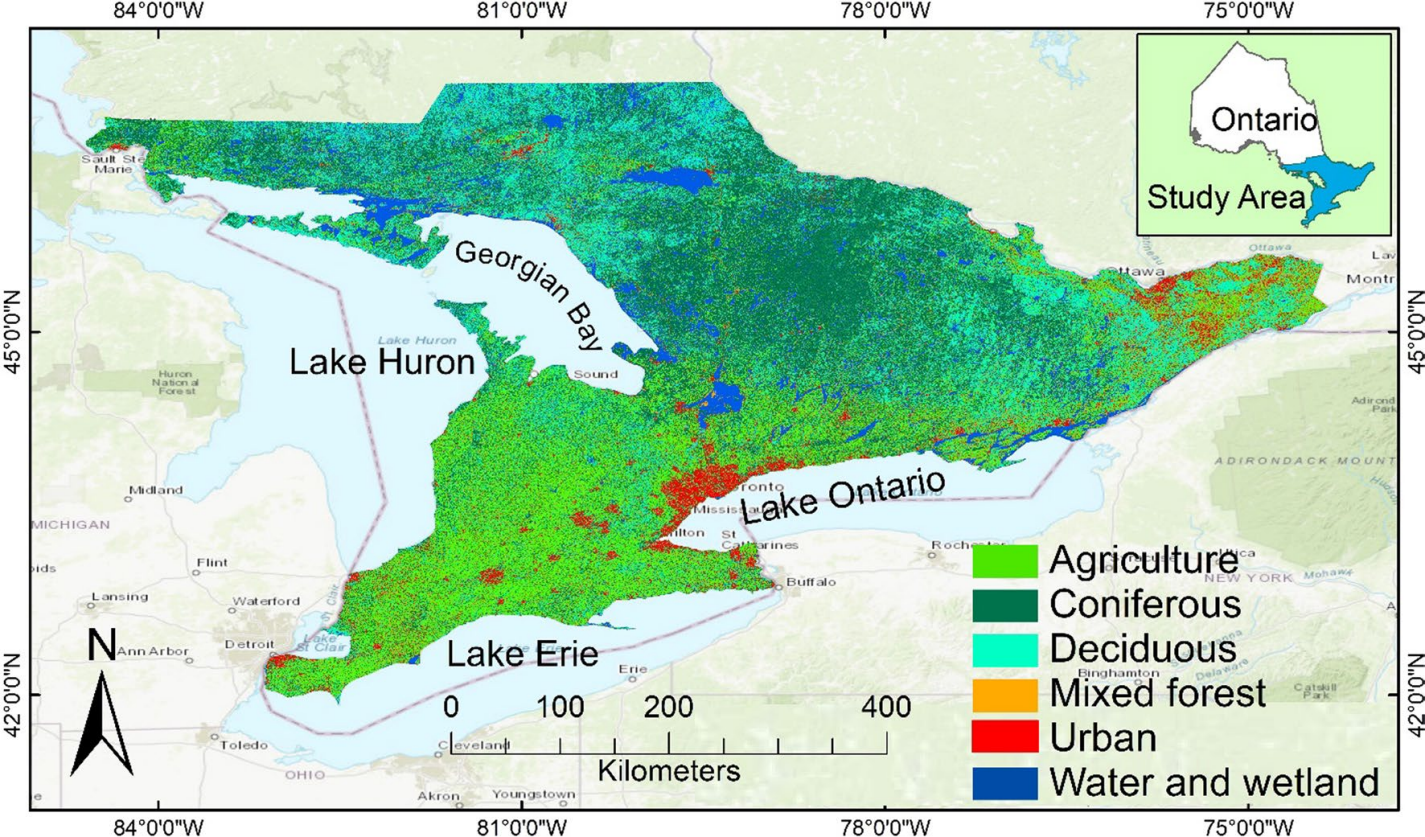
Study area map. The LULC map was generated by machine learning-based Google Earth Engine (GEE) using Sentinel-2 remote sensing data from the composite images of the growing season of 2020

In Eastern North America, spongy moth outbreaks have occurred roughly every seven to ten years with the past major or significant infestations recorded in 1981, 1985, 1991, 2002, 2008 and 2021 (OMNRF 2024; ONDMNRF 2021). Since 1970, it is estimated that over 30 million hectares of forest have experienced defoliation due to spongy moth infestation (De Beurs and Townsend 2008; Hajek et al. 2021). The spongy moth outbreak of 2021 was the largest on record in the region where almost 1.78 million hectares of forests were impacted in the province of Ontario, Canada (Fig. 1) and 2.5 million hectares affected in the United States (USDA 2023; OMNRF 2024). In Ontario, 17,797 km^2^ forest area was severely impacted by the infestation (OMNRF 2024). The large-scale 2021 spongy moth defoliation severely impacted C sequestration capabilities of forest ecosystems in both Canada and USA and posed a considerable challenge for the health and growth of forests (Chung et al. 2021). With about 595 million hectares of non-affected forests in North America that are climatically suitable habitats for spongy moth expansion, future outbreaks may potentially pose a major challenge for forest growth, health and C uptake in the region (Gray 2004; Kalamandeen et al. 2023). Therefore, there is a need to develop effective forest monitoring and management strategies and develop integrated methods to quantify the loss of C caused by these infestations, which are expected to become more widespread, intense and frequent in future due to climate change (De Beurs and Townsend 2008; Harvey et al. 2022).

Remote sensing techniques have been effectively employed for estimating spongy moth defoliation areas since the mid-1980s utilizing Earth observatory satellite imagery from platforms such as Landsat (Williams et al. 1985; Joria et al. 1991; White et al. 2017), SPOT-1 (Ciesla et al. 1989), and MODIS (De Beurs and Townsend 2008). These satellite systems typically classify regions impacted by spongy moth infestations into different categories, including light, moderate, and heavy defoliation, while also identifying regions of healthy forests (Williams et al. 1985; Ciesla et al. 1989; Joria et al. 1991; Kovalev et al. 2023). However, the precise categorization of the intensity of spongy moth infestation has been challenging, primarily due to the shorter duration of spongy moth outbreak and low or moderate resolution of satellite imagery (e.g. from MODIS, SPOT, and Landsat Satellites). Recent advances in high-resolution remote sensing techniques have significantly improved the accuracy of remote sensing images, enabling not only the detection of defoliation areas but also providing capabilities for the precise measurements of the extent of these events and quantifying defoliation impacts on C sequestration (Townsend et al. 2004; Kovalev et al. 2023). It allows systematic assessment of the influence of spongy moth infestations on forest ecosystems and their C balances.

Sentinel-2A and 2B satellites provide high-resolution (10 × 10 m^2^) images that are very suitable for monitoring insect infestation such as spongy moth defoliation and for quantifying forest C losses through the exploration of vegetation indices (VIs), and estimation of GPP (Hussain et al. 2024). Several studies in the literature have successfully estimated infestation impact on forest growth and health by utilizing VIs such as the normalized difference vegetation index (NDVI) and the enhanced vegetation index (EVI) (Carter and Knapp 2001; Fraser & Latifovic 2005; Eklundh et al. 2009). However, studies focusing on the quantification of the effects of insect defoliation on forest C dynamics has been limited (De Beurs and Townsend 2008; Senf et al. 2017; Kovalev et al. 2023).

The primary aim of this study is to determine the impact of 2021 spongy moth (*Lymantria dispar*) infestations on forest growth and productivity in the Great Lake region in Canada using high-resolution (10 × 10 m^2^) Sentinel-2 satellite remote sensing data and eddy covariance (EC) flux observations from 2020 to 2022. The specific objectives of this study are to: (i) estimate seasonal variations and trends in the leaf area index (LAI) using high resolution remote sensing data; (ii) determine forest photosynthetic uptake and gross primary productivity (GPP) using observed eddy covariance flux and remote sensing data; and (iii) quantify carbon (C) losses across the region because of this wide spread and server spongy moth infestation. To delineate distinct vegetation categories within the study area, the study employed a machine learning-based land use/land cover (LULC) classification scheme using Sentinel-2 data in the Google Earth Engine (GEE) platform. An examination of the suitability of utilizing LAI to measure the biomass and GPP of various affected vegetation cover types across the region was also conducted. These assessments will contribute to the development of sustainable forest management strategies and help to achieve net zero carbon goals through nature-based climate change solutions.

## Materials and methods

### Study area

The study area covers a region from 75° W to 84° W longitude and 42° N to 48° N latitude, situated along the shores of Lake Ontario, Lake Erie, and Lake Huron, encompassing approximately 178,000 km^2^ in southern and central Ontario, Canada (Fig. 1). Much of this area is part of Great Lakes-St. Lawrence forest which is dominated by different ages of hardwood forests including a variety of tree species such as sugar maple (*Acer saccharum*), red maple (*Acer rubrum*), white oak (*Quercus alba*), red oak (*Quercus rubra*), yellow birch (*Betula alleghaniensis*), basswood (*Tilia americana*), white pine (*Pinus strobus*), red pine (*Pinus resinosa*), Eastern hemlock (*Tsuga canadensis*) and white cedar (*Thuja occidentalis*). Deciduous, conifer and mixed forests cover up to 62% land of this area. The southern latitudes of the study area are dominated by cropland such as corn, soybean, and forage for livestock production, as well as deciduous forests which cover about 10% of the area (OMNRF 2024). The remaining land is categorized as primary wetlands or urban areas. The northern parts of study area is part of the Boreal forest and the Georgian Bay lowlands forest, while the central and southern forests are also characterized as Carolinian forests. The southern region is more conducive to agriculture, more densely populated, and urbanized. In contrast, the central and northern regions of the study areas are mountainous terrain covered with forests and have a relatively untouched environment (Baldwin et al. 2000; Shah et al. 2022).

The climate of the study area is characterized as cool continental, which is influenced by regional factors due to area proximity to the Great Lakes. The mean annual precipitation of 786 mm year^−1^ based on observations recorded at the Toronto Pearson Airport Weather Station during the normal climate period from 1991 to 2020 (Environment and Climate Change Canada 2023). 14% of the precipitation fell as snow. The mean annual temperature varies across the region depending on latitude, with mean annual temperature of 8.2 °C from 1991 to 2020 at the Toronto Pearson airport weather station (Environment and Climate Change Canada 2023). Additionally, mean temperature during the growing season fluctuates between 15 and 30 °C (Wazneh et al. 2017).

### Remote sensing and observed eddy covariance flux datasets

Sentinel-2A and Sentinel-2B (S2) satellites of the COPERNICUS satellite systems of the European Union's earth observation program (Drusch et al. 2012) provide high-resolution datasets for terrestrial ecosystem monitoring (Löw & Koukal 2020). The Sentinel-2 multispectral instrument (MSI) system delivers 13 spectral bands, including 10 × 10 m^2^ spatial resolution of visible and near-infrared (NIR) and 20 × 20 m^2^ spatial resolution of short-wave infrared (SWIR) spectrum with up to five-day revisiting time (Drusch et al. 2012; Sun et al. 2021). This study used Sentinel-2 data to calculate vegetation indices (VIs) such as normalized difference vegetation index (NDVI), and leaf area index (LAI) for biomass estimation. Sentinel-2 satellite datasets were downloaded from https://earthexplorer.usgs.gov/. Sentinel-2 (S2) data was also used to estimate GPP while utilizing radiative model and observed eddy covariance (EC) flux data.

**Fig. 2:**
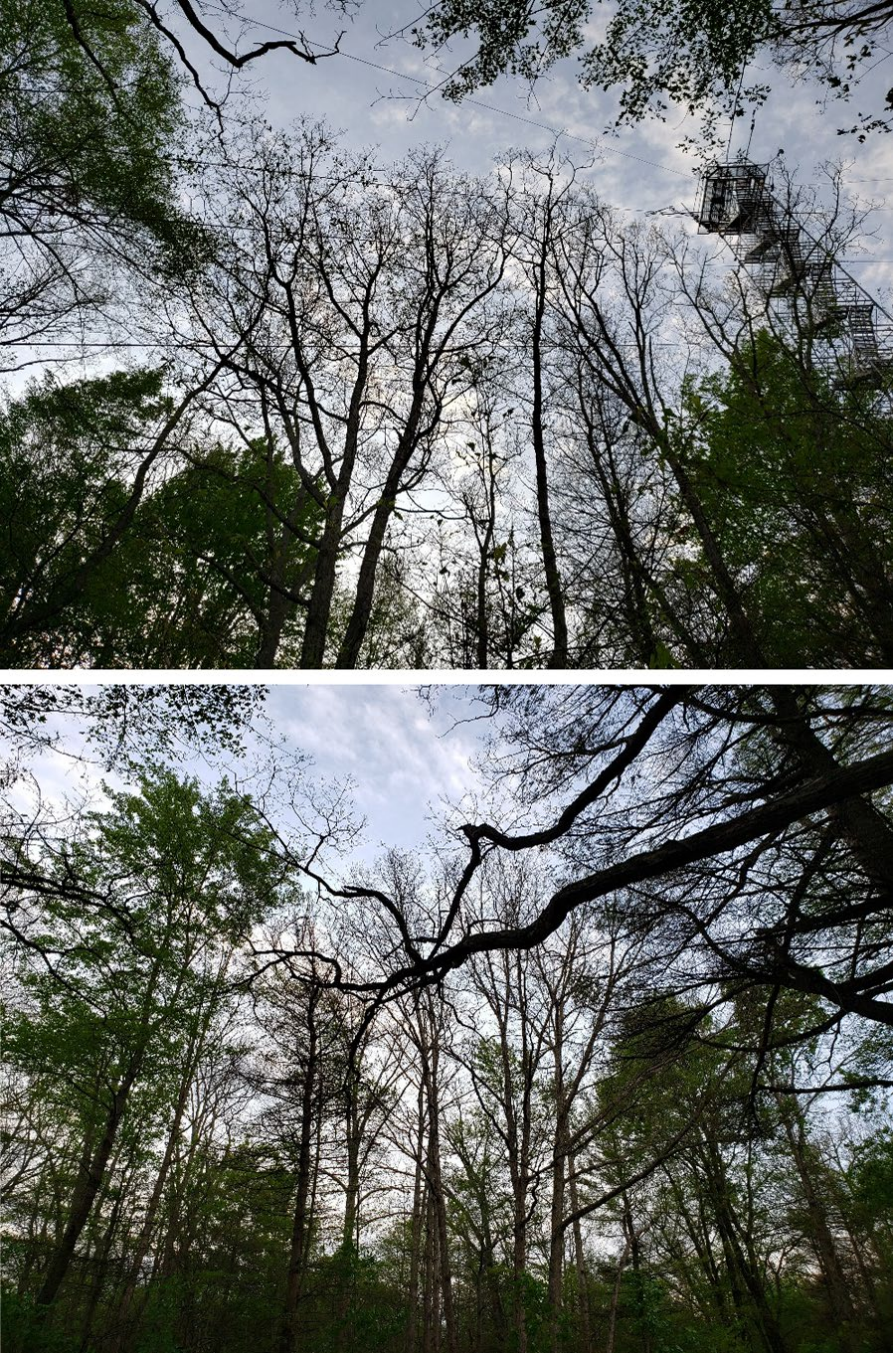
Defoliated trees due to spongy moth infestation at the Turkey Point Environmental Observatory’s deciduous forest site on 21 June 2021

The observed EC flux data were obtained from Turkey Point Environmental Observatory (Arain et al. 2022; Beamesderfer et al. 2020; Latifovic and Arain 2024). This site is known as the Canadian Turkey Point deciduous forest site (CA-TPD) and is associated with of the Global Water Futures Observatory Program, Ameriflux and Global Fluxnet network (Arain 2018). Although EC flux and meteorological variable has been continuously measured at this site since 2012, CO_2_ fluxes for three years, i.e. 2020 (pre-infestation), 2021 (infestation) and 2022 (post infestation) were used in the analysis presented in this study. 2021 spongy month infestation was quite severe at our forest site where majority of deciduous trees were defoliated as shown in Fig. 2 and further discussed in Latifovic and Arain (2024). The quality control of EC flux and meteorological data was conducted utilizing the Biometeorological Analysis, Collection, and Organizational Node (BACON) software, which was developed by our lab (Brodeur 2014). Outliers within the dataset were detected and eliminated through the BACON software and small gaps in the dataset were filled through linear interpolation from adjacent forest sites CA-TP3 and CA-TP4. Further details of EC fluxes and meteorological measurements, data gap filling and partitioning of observed CO_2_ flux in ecosystem respiration and GPP are given in Latifovic and Arain (2024). In addition, no forest management activity had taken place at the forest in recent years.

### Land use and land cover (LULC) classification

The GEE platform's machine-learning approach was utilized to create cloud-free Sentinel-2 data for the LULC analysis (Nasiri et al. 2022). The GEE cloud computing approach was utilized to collect images and process data for growing season of 2020 (Fig. 1). GEE-based machine learning classifier, support vector machine (SVM) was used to classify six primary land cover categories, namely water bodies, urban areas, agricultural land, coniferous forest, deciduous forest and mixed forest (Sheykhmousa et al. 2020). Each land cover category was assessed using 650 ground point samples to extract per-band pixel values from the Sentinel-2 dataset, ensuring that the data used had minimum cloud cover (less than 5%). The evaluation of classification accuracy provided a comparison between LULC classes derived from the training point and data obtained during the testing phase (Nasiri et al. 2022), which involved a total of 3900 ground point samples. This accuracy assessment was performed using confusion matrices (Table 1). The overall accuracy based on these confusion matrices was 95.7%.

**Table 1:** Confusion matrices-based accuracy assessment of land use and land cover (LULC) classification

Class	Water body	Unban area	Agricultural land	Deciduous forest	Coniferous forest	Mixed forest	Producer accuracy (%)	User accuracy (%)
Water body	627	1	0	0	0	2	98.4	99.4
Unban area	1	617	0	6	2	2	96.7	98.0
Agricultural land	3	1	622	8	6	10	97.2	96.2
Deciduous forest	3	9	12	606	22	12	96.4	94.2
Coniferous forest	4	12	7	16	610	11	95.5	94.7
Mixed forest	12	10	9	14	10	613	94.7	94.1

Overall accuracy: 95.7%

The analysis revealed that coniferous forests occupied the largest land area, covering 43,017 km^2^, which represents 24.29% of the total studied area. Agriculture was the second-largest land cover category, covering 42,294 km^2^, accounting for 23.88% of the total area. Deciduous forests covered 36,574 km^2^, constituting 20.64% of the total area and mixed forests occupied 23,936 km^2^, making up to 13.51% of the total area. Additionally, water bodies and wetlands covered 13,053 km^2^, covering 7.37% of the total area, while the urban areas occupied 19,256 km^2^, covering 10.87% of the total area.

### Retrieval of leaf area index (LAI)

LAI was calculated using the Sentinel-2 data and the PROSAIL model which is the combination of PROSPECT (Jacquemoud and Baret 1990; Feret et al. 2008) and SAIL model (Verhoef 1984). The PROSPECT model provides leaf optical properties and the SAIL model provides plant canopy reflectance (Sun et al. 2021). The PROSPECT model measures leaf hemispherical reflectance and transmittance to define leaf optical elements at 400–2500 nm through six input parameters: leaf structure parameter (N, unitless), leaf chlorophyll content (C_ab_), carotenoid content (C_ar_), brown pigment content (C_brown_), equivalent water thickness (C_w_) and dry matter content (C_m_) (Xu et al. 2019). The SAIL model calculates canopy reflectance as a function of leaf optical elements obtained from PROSPECT and six input parameters: leaf inclination distribution function (LIDF), LAI, hot spot parameter (hspot), solar zenith angle (tts), view zenith angle (tto), relative azimuth angle (psi) (Sun et al. 2021). All input parameters for the PROSAIL model are shown in Table 2.

**Table 2:** Input parameters for the PROSAIL model. The fixed value is used in this study

Model	Input Parameters	Symbol	Unit	Range	Fixed value
PROSPECT	Leaf structure	N	Dimensionless	1.5–3.0	1.5
	Chlorophyll content	C_ab_	µg cm^−2^	10–80	40
	Carotenoid content	C_ar_	µg cm^−2^		10
	Brown pigment	C_brown_	Arbitrary units		0
	Equivalent water thickness	C_w_	cm		0.01
	Dry matter content	C_m_	g cm^−2^		0.009
SAIL	Leaf inclination distribution function	LIDF	Shape	Spherical	Spherical
		LIDFa	Slope	− 1 to 1	− 0.35
		LIDFb	Kind of distortion	− 1 to 1	− 0.15
	Leaf area index	LAI	m^2^ m^–^^2^	0–8	
	Hot spot parameter	hspot	m/m	0.03–0.1	0.01
	Solar zenith angle	tts	(°)	20–70	30
	View zenith angle	tto	(°)	0–30	10
	Relative azimuth angle	psi	(°)		0

The spectral response function for Sentinel-2 satellite data were used from band effective reflectance. The band reflectance was calculated based on the measured canopy hyperspectral reflectance and simulated reflectance from the PROSAIL model. The band reflectance was calculated by Wang et al. (2015) as follows:

1 $$\rho_{s} \left( \lambda \right) = \frac{{\mathop \smallint \nolimits_{{\lambda_{min} }}^{{\lambda_{max} }} \rho_{s} \left( {\lambda_{i} } \right)\psi \left( {\lambda_{i} } \right)d\lambda }}{{\mathop \smallint \nolimits_{{\lambda_{min} }}^{{\lambda_{max} }} \psi \left( {\lambda_{i} } \right)d\lambda }}$$

Its derivative follows as:

2 $$\rho_{s} \left( \lambda \right) = \frac{{\mathop \smallint \nolimits_{400}^{2500} \rho_{s} \left( {\lambda_{i} } \right)\psi \left( {\lambda_{i} } \right)d\lambda }}{{\mathop \smallint \nolimits_{400}^{2500} \psi \left( {\lambda_{i} } \right)d\lambda }} \approx \frac{{\mathop \sum \nolimits_{400}^{2500} \rho_{s} \left( {\lambda_{i} } \right)\psi \left( {\lambda_{i} } \right)}}{{\mathop \sum \nolimits_{400}^{2500} \psi \left( {\lambda_{i} } \right)}}$$

where ${\rho }_{s}(\lambda )$ is the simulated band reflectance of the sensor, ${\rho }_{s}({\lambda }_{i})$ is the simulated reflectance of the PROSAIL model, which is coded in MATLAB (MathWorks Inc.). ${\lambda }_{min}$ is equal to 400 nm, the minimum value of wavelength limit and ${\lambda }_{max}$ is 2500, the maximum value of the wavelength limit and $\psi \left({\lambda }_{i}\right)$ is the spectral response coefficient of Sentinel-2.

### Remote sensing-based gross primary productivity (GPP) estimation

GPP was estimated using the Sentinel-2-based light use efficiency (LUE) model to quantify the CO_2_ uptake from different vegetation cover types. LUE model has the empirical capability to estimate GPP (Zhang et al. 2017; Sun et al. 2019) using remote sensing data. Observed air temperature (T_a_) and photosynthetically active radiation (PAR) data were used with satellite data in the LUE model to calculate GPP (Hussain et al. 2024). Following equations were used as part of the LUE model (Table 3).

**Table 3:** Equations used to calculate ecosystem properties

Variables	Equation	References
GPP	GPP = APAR_chl_ × ε_g_	Monteith (1972)
	APAR_chl_ = PAR × fPAR_chl_	Xiao et al. (2004)
	fPARchl = (EVI − 0.1) × 1.25	Zhang et al. (2017)
LUE	ε_g_ = ε_0_ × T_scalar_ × W_scalar_	Zhang et al. (2017)
	${{\text{T}}}_{{\text{scalar}}}=\frac{({\text{Ta}}-{{\text{T}}}_{{\text{max}}})\times ({\text{Ta}}-{{\text{T}}}_{{\text{min}}})}{\left({\text{T}}-{{\text{T}}}_{{\text{max}}}\right)\times \left({\text{Ta}}-{{\text{T}}}_{{\text{min}}}\right)-({\text{Ta}}-{{{\text{T}}}_{{\text{opt}}})}^{2}}$	Zhang et al. (2016)
	${{\text{W}}}_{{\text{scalar}}}=\frac{1+{\text{LSWI}}}{1+{{\text{LSWI}}}_{{\text{max}}}}$	Zhang et al. (2016)
Indices	NDVI = (R_NIR_ − R_Red_)/(R_NIR_ + R_Red_)	Rouse et al. (1974)
	${\text{EVI}}=2.5 \times \frac{{{\text{R}}}_{{\text{NIR}}}-{{\text{R}}}_{{\text{Red}}}}{{{\text{R}}}_{{\text{NIR}}}+6{ \times \mathrm{ R}}_{{\text{Red}}}-7.5 \times {{\text{R}}}_{{\text{Blue}}}+1}$	Huete et al. (2002)
	LSWI = (R_NIR_ − R_SWIR_)/(R_NIR_ + R_SWIR_)	Xiao et al. (2004)

**Fig. 3:**
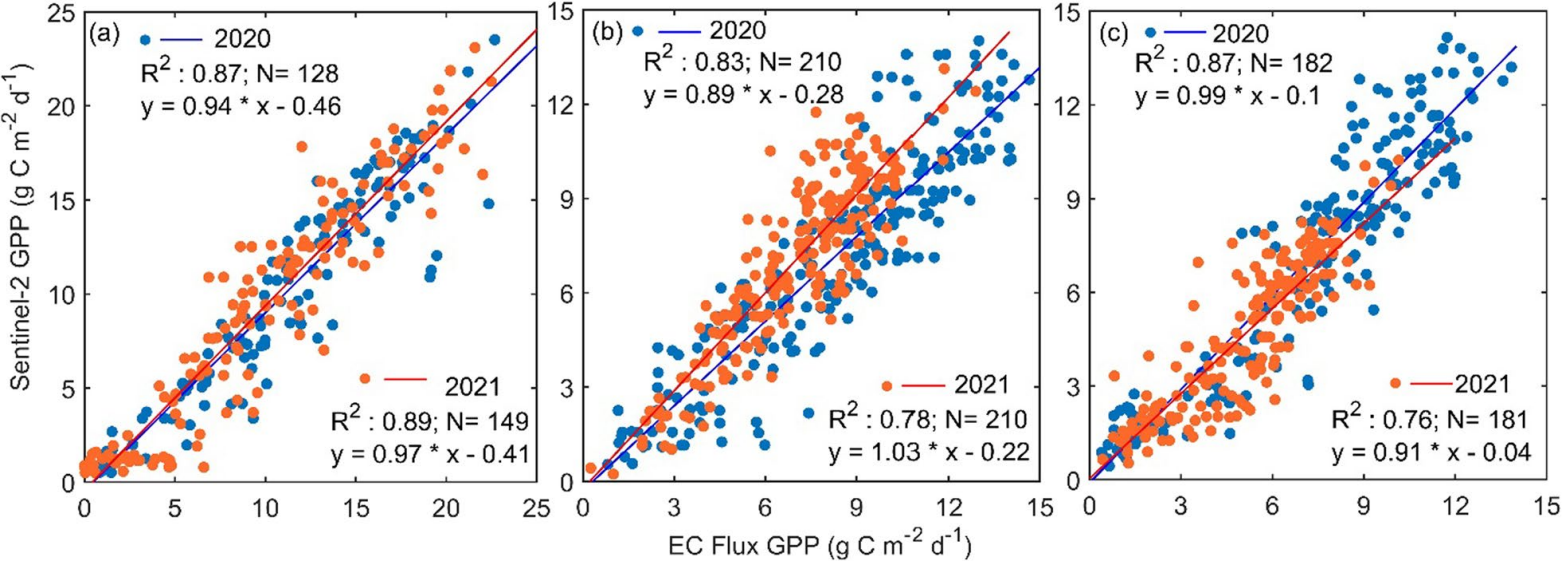
The relationship between satellite-derived and eddy covariance (EC) flux tower based observed daily gross primary productivity (GPP) values for **a** agricultural lands, **b** conifer forests and **c** deciduous forests, respectively from 2020 to 2021

In Table 3, APAR_chl_ is absorbed photosynthetically active radiation (PAR); fPAR_chl_ is the fraction of PAR estimated by chlorophyll or linear function of EVI, which is modified following Xiao et al. (2004). 0.1 and 1.25 are constants to adjust for vegetated land and were validated from solar-induced chlorophyll fluorescence (SIF); ε_g_ is the light use efficiency (LUE), ε_0_ is the apparent quantum yield or maximum light use efficiency [µmol CO_2_ per µmol photosynthetic photon flux density (PPFD)]; T_scalar_, W_scalar_ are the downward-parameter scalars for the effects of temperature and water respectively on LUE by C3/C4 photosynthesis pathways; Ta, ${{\text{T}}}_{{\text{min}}}$, ${{\text{T}}}_{{\text{max}}} ,$ and ${{\text{T}}}_{{\text{opt}}}$ refer to the mean, minimum, maximum, and optimum temperature for photosynthesis, respectively; LSWI is the land surface water index. Model estimated daily GPP values were compared with the observed GPP values for 2020 and 2021 as shown in Fig. 3. There was a strong correlation between satellite-derived and observed daily GPP values for agricultural lands, conifer forests and deciduous forests, respectively (Fig. 3a–c).

### Statistical analysis

Weighted double logistic (WDL) function was used to fit the daily time series of VIs as described in Yang et al. (2019). WDL consists of two logistic functions based on the vegetation growth activity, including the growing part (*f*_1_) and the declining part (*f*_2_) to set the model parameters which can provide the daily time series using following equations (Yang et al. 2019).

3 $$y = {\text{f}}_{{1}} + {\text{f}}_{{2}} + {\text{e}}$$

4 $${\text{f}}_{{1}} = \frac{{c_{1} }}{{1 + e^{{a_{1} + b_{1} t}} }} + d_{1}$$

5 $${\text{f}}_{{2}} = \frac{{c_{2} }}{{1 + e^{{a_{2} + b_{2} t}} }} + d_{2}$$

6 $${\text{e}} = max\left( {c_{1} + d_{1} ,c_{2} + d_{2} } \right)$$

where *y* is the time series of variable, *d* and *c* + *d* denote the minimum value *(min(f))* and maximum value *(max(f))*, respectively; *c* indicates the local amplitude; and *a* and *b* determine the shape and slope of the logistic function, respectively. The subscripts 1 and 2 identify the parameters of the growing and declining parts, respectively. In the retrieval of these unknown parameters, the initial *d* and *c* are assigned as *min(f)* and *max(f)* − *min(f)*, respectively. Thus, the principal problem is to derive parameters *a* and *b*. Considering the different weights of each of the data points, we transformed the non-linear fitting problem into *a* linear one by *a* function transformation as *a*_1_ + *b*_1_*t* = ln(*c*_1_*f*_1_ − *d*_1_ − 1). Furthermore, the WLS method is applied to solve the analytic expression of the logistic function for each part (*f*_1_ and *f*_2_).

We also utilize standardized anomalies to understand temporal variations and deviations from normal growth trends over the study period. We calculated these anomalies by subtracting the mean GPP during three growing periods from the daily GPP values and then dividing it by the standard deviation observed over the same periods. These calculations followed Eqs. 7 and 8 as shown by Zhao et al. (2022).

7 $$y_{sd} = \frac{{y_{d} - \overline{{y_{d} }} }}{ \sigma }$$

8 $$y_{d} = x - \overline{x}$$

where ${y}_{sd}$ is standardized anomaly, ${y}_{d}$ is daily anomaly and $x$ is daily GPP and $\overline{x }$ is the three-year mean GPP estimated from Sentinel-2.

## Results

### Climatic conditions

**Fig. 4:**
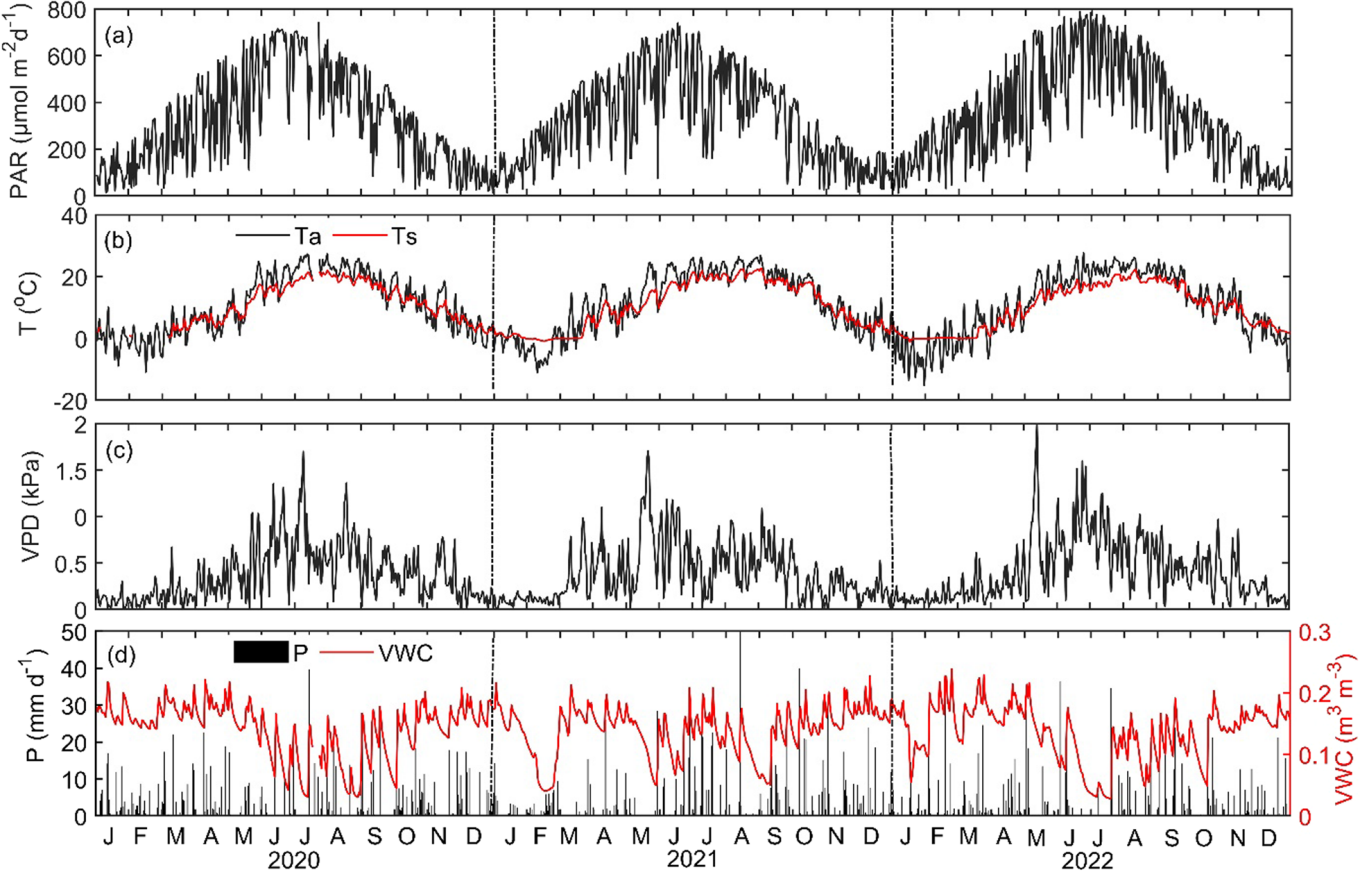
Daily mean values of **a** photosynthetically active radiation (PAR), **b** air temperature (Ta) and soil temperature (Ts) at 5 cm depth, **c** vapor pressure deficit (VPD), **d** precipitation (P) and volumetric water content (VWC) from 2020 to 2022

**Fig. 5:**
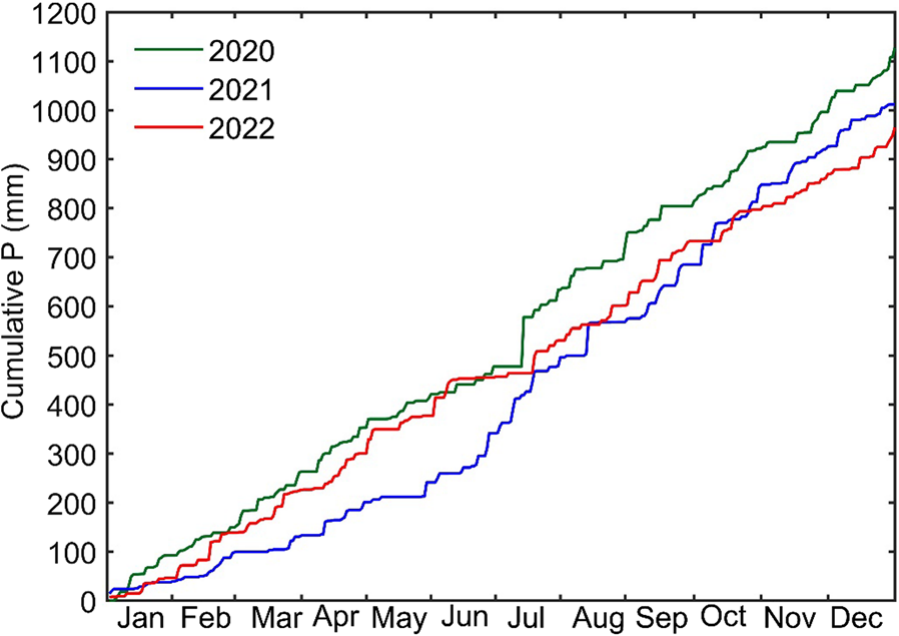
Daily cumulative precipitation (P) from 2020 to 2022

The meteorological variables measured at our site from 2020 to 2022 are shown in Figs. 4 and 5. The mean annual Ta was 10.6, 11.3, and 10.6 °C for 2020, 2021, and 2022, respectively. The daily maximum Ta was observed during July–August periods, while minimum Ta values were observed during January–February, reflecting the typical seasonal patterns in the Great Lakes region (Fig. 4b). At the same time, Ts was 9.7, 10.3, and 9.6 °C. Temporal variability in Ts closely followed the temporal variability of Ta, with a correlation coefficient of 0.89 (*P* < 0.001). Additionally, photosynthetically active radiation (PAR) exhibited similar patterns to temperature variations (Fig. 4a, b), with respective daily values of 317, 321, and 343 μmol m^−2^ d^−1^ for 2020, 2021, and 2022.

The daily mean values of VPD were 0.37, 0.38, and 0.38 kPa for 2020, 2021, and 2022, respectively. The similarity between VPD values across the years indicates overall relatively stable atmospheric moisture conditions during the study period. Additionally, VWC during the same period was 0.11, 0.12, and 0.11 m^3^ m^−3^. The temporal variations in VWC reflected changes in soil moisture following large precipitation events throughout the year (Fig. 4d). The annual total precipitation values were 1127, 1009, and 960 mm for 2020, 2021, and 2022, respectively (Fig. 5). 2021 showed a dry period with low precipitation values in 2021 from early March to mid-June. This dry and rain free period in early parts of the growing season in 2021 may have helped spongy moth to establish and thrive. Overall, observed meteorological conditions during the study period showed similarities with long-term observed weather conditions at this site.

### Dynamics of remote sensing-based leaf area index (LAI)

**Fig. 6:**
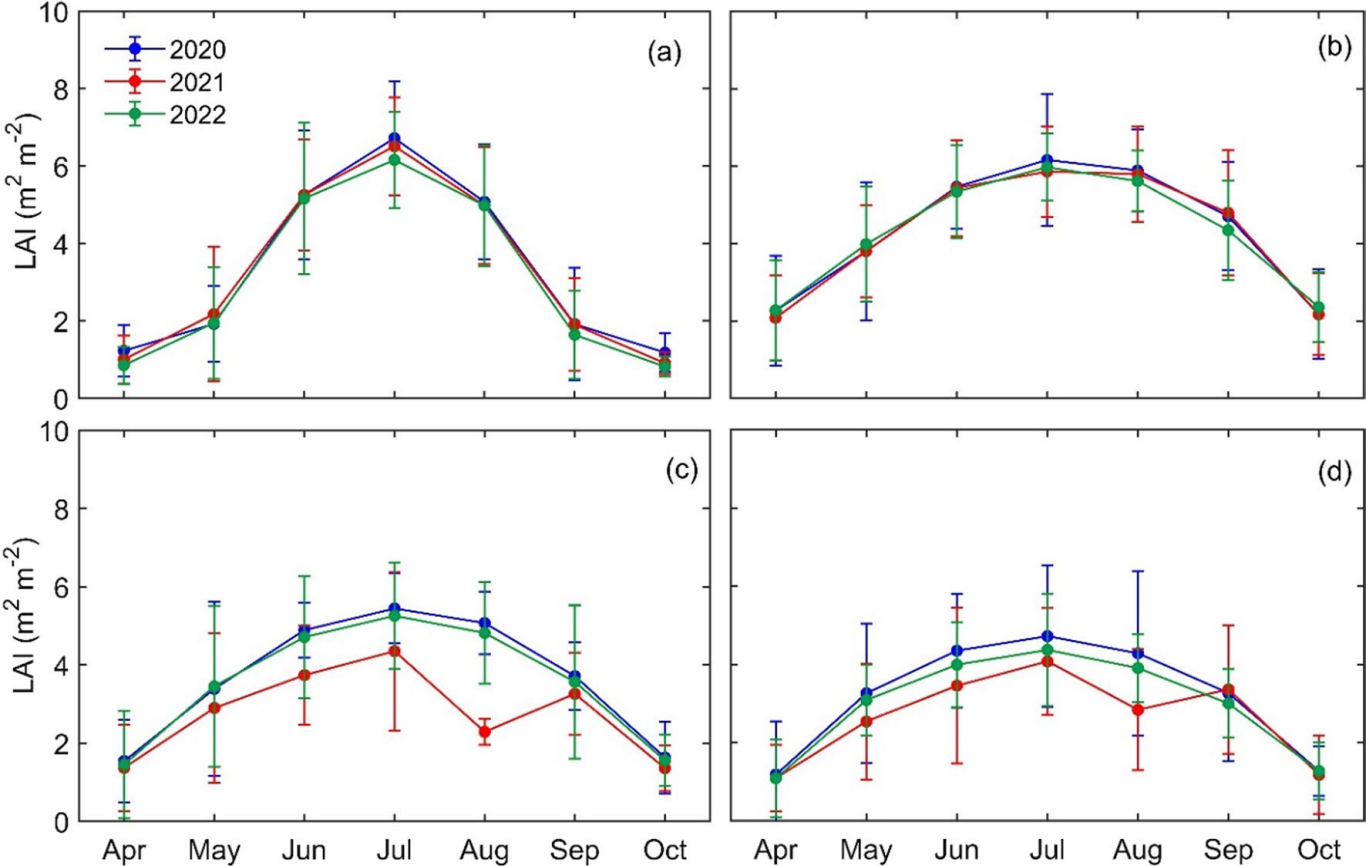
Monthly mean leaf area index (LAI) values over the study area for **a** agricultural lands, **b** conifer forests, **c** deciduous forests and **d** mixed forests from 2020 to 2022

Remote sensing-based monthly mean LAI values for major land cover types including deciduous, conifer and mixed forests and agricultural lands over the growing season are shown in Fig. 6. Deciduous forests had mean LAI value of 3.66 (± 1.6), 2.74 (± 1.1), and 3.53 (± 1.5) m^2^ m^−2^, conifer forests had LAI value of 4.34 (± 1.6), 4.28 (± 1.6), and 4.26 (± 1.5) m^2^ m^−2^ and mixed forest had LAI value of 3.18 (± 1.4), 2.64 (± 1.1), and 2.94 (± 1.3) m^2^ m^−2^ for 2020, 2021, and 2022, respectively. Mean LAI values for agricultural lands were 3.31 (± 2.2), 3.25 (± 2.3), and 3.11 (± 2.2) m^2^ m^−2^ for respective years. The highest LAI values were observed for agricultural lands and conifer forests in July, followed by deciduous and mixed forests. These satellite-derived LAI values showed a large decline for deciduous and mixed forests in 2021, when these forests were impacted by spongy moth infestations (Fig. 6c, d).

Mean LAI values for deciduous and mixed forests declined by 25 (22)% and 17 (10)% in comparison to the pre-infestation (post-infestation) values recorded in 2020 (2022). LAI values recovered to almost normal levels in 2022 for deciduous forest after the infestation, while LAI for mixed forest showed relatively lower recovery values.

### Impact of spongy moth infestation on gross primary productivity (GPP)

**Fig. 7:**
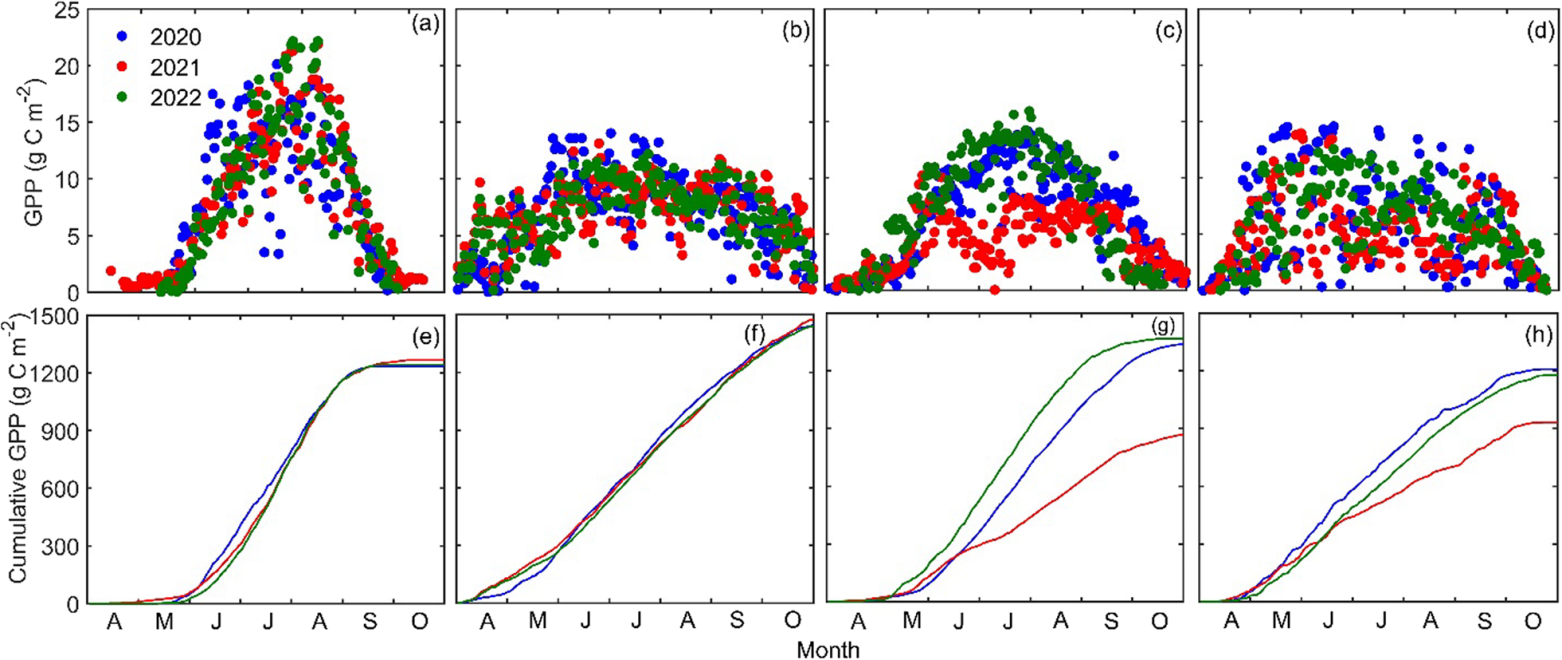
Daily gross primary productivity (GPP, g C m^−2^ d^−1^) for **a** agricultural lands, **b** conifer forests, **c** deciduous forests and **d** mixed forests, respectively, from 2020 to 2022. Similarly, cumulative GPP values over the growing season for **e** agricultural lands, **f** conifer forests, **g** deciduous forests and **h** mixed forests, respectively from 2020 to 2022

The satellite derived daily GPP values showed similar trends as observed for LAI, with much lower daily GPP values for deciduous and mixed forests in 2021 due to spongy moth infestation (Fig. 7). In deciduous forests, photosynthetic C uptake usually started in mid-May and peaked in July with typical maximum daily GPP values of about 14 to 16 g C m^−2^ d^−1^. However, in 2021, GPP values rapidly declined at the start of June when spongy moth defoliation intensified. Daily GPP values reached as low as 3.0 g C m^−2^ d^−1^ in July in 2021. Similar low GPP values were also observed for mixed forests. GPP saw a rebound in late July and August when the short-lived spongy moth infestation started to end due to transformation of leaf-eating larvae (caterpillars) to pupa and adult stages. In addition, these decreasing trends of GPP were well aligned with the spongy moth life cycle, where the late caterpillar stage occurs from mid-May to the end of July, causing extensive leaf damage. However, after this period, daily GPP values showed some recovery but only reached up to 7 to 8 g C m^−2^ d^−1^ before the usual autumn photosynthetic decline started to take effect in late September. In general, rebounded daily GPP values were even lower for mixed forests due to the combined effects of infestation for deciduous forests and usual seasonal low soil moisture from late July to August in the region, which typically causes lower GPP values in conifer trees. However, overall the soil moisture was sufficient for ecosystem production in 2021 (Fig. 4d). In 2020 and 2021, the active period of growth for deciduous forests ended by the end of October, while in 2022 deciduous forests experienced an earlier end of growing season (Fig. 7c).

In contrast, photosynthetic C uptake in coniferous forests began earlier in April as compared to other vegetation types and continued until the end of October. The maximum daily GPP in conifer forests was observed in June, with maximum daily GPP values of about 10 to 14 g C m^−2^ d^−1^. In agricultural lands, daily GPP was almost zero in April but it started to increase in mid-May and peaked in July and August, with maximum daily GPP values reaching about 20 to 23 g C m^−2^ d^−1^ (Fig. 7a).

**Fig. 8:**
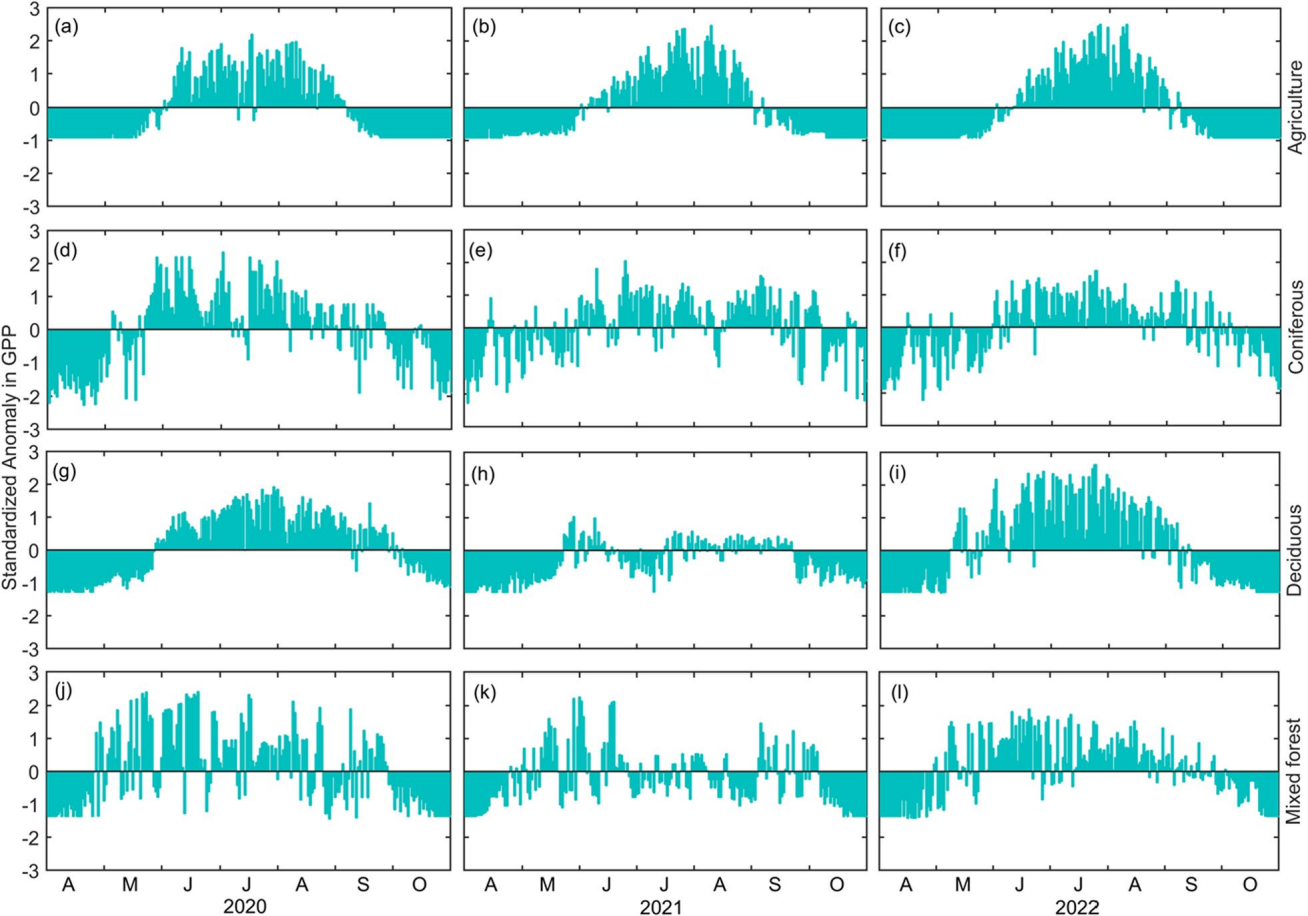
The daily standardized anomaly in gross primary productivity (GPP, g C m^−2^ d^−1^) for agricultural lands (**a**, **b**, **c**), conifer forests (**d**, **e**, **f**), deciduous forests (**g**, **h**, **i**) and mixed forests (**j**, **k**, **l**) for 2020, 2021 and 2022

These trends were also clearly shown in the standardized daily GPP anomaly values, where GPP in deciduous and mixed forests showed large decline, while GPP in conifer forests and agricultural lands were not impacted (Fig. 8). In 2022, the forest appeared to be fully recovered with a notable increase in both the daily mean and seasonal total GPP values as compared to 2021.

Overall, growing season mean daily GPP values in deciduous forests were 6.83 (± 4.1), 4.43 (± 2.5), and 7.77 (± 5.4) g C m^−2^ d^−1^ for 2020, 2021 and 2022, respectively. Corresponding GPP values for coniferous forests were 6.87 (± 3.5), 7.10 (± 2.7), and 6.86 (± 2.7) g C m^−2^ d^−1^ and for mixed forests were 6.45 (± 4.2) g C m^−2^ d^−1^, 4.81 (± 2.2) g C m^−2^ d^−1^, and 6.12 (± 2.3) g C m^−2^ d^−1^. Agricultural lands had growing season mean daily GPP values of 9.65 (± 5.4), 8.45 (± 6.1), and 9.55 (± 6.2) g C m^−2^ d^−1^ in 2020, 2021, and 2022, respectively (Fig. 7; Table 4). The highest cumulative GPP values over the growing season were observed in the coniferous forest in all three years, followed by deciduous forests, agricultural lands, and mixed forests (Fig. 7e–h and Table 4). Maximum GPP estimates for conifer forests highlighted their optimum photosynthetic activity and proficiency for C uptake. Deciduous forests had total growing season GPP values of 1338, 869, and 1367 g C m^−2^ in 2020, 2021 and 2022, respectively, while coniferous forests photosynthesized 1443, 1475, and 1438 g C m^−2^ and mixed forests exhibited GPP values of 1208, 932, and 1175 g C m^−2^ for the same years (Fig. 7e–h and Table 4). Agricultural lands showed cumulative GPP values of 1235, 1266, and 1241 g C m^−2^ over the same period (Fig. 7e–h and Table 4).

**Table 4:** Mean daily gross primary productivity (GPP) of different vegetation types in growing season (g C m^−2^)

Vegetation type	2020		2021		2022	
	Daily mean	Seasonal total	Daily mean	Seasonal total	Daily mean	Seasonal total
Agriculture land	9.65 ± 5.4	1235	8.45 ± 6.1	1266	9.55 ± 6.2	1242
Conifer forest	6.87 ± 3.5	1443	7.10 ± 2.7	1475	6.86 ± 2.7	1438
Deciduous forest	6.83 ± 4.1	1338	4.43 ± 2.5	868	7.77 ± 5.4	1367
Mixed forest	6.45 ± 4.2	1208	4.81 ± 2.2	932	6.12 ± 2.3	1175
Mean	7.45	1306	6.20	1135	7.58	1305

**Fig. 9:**
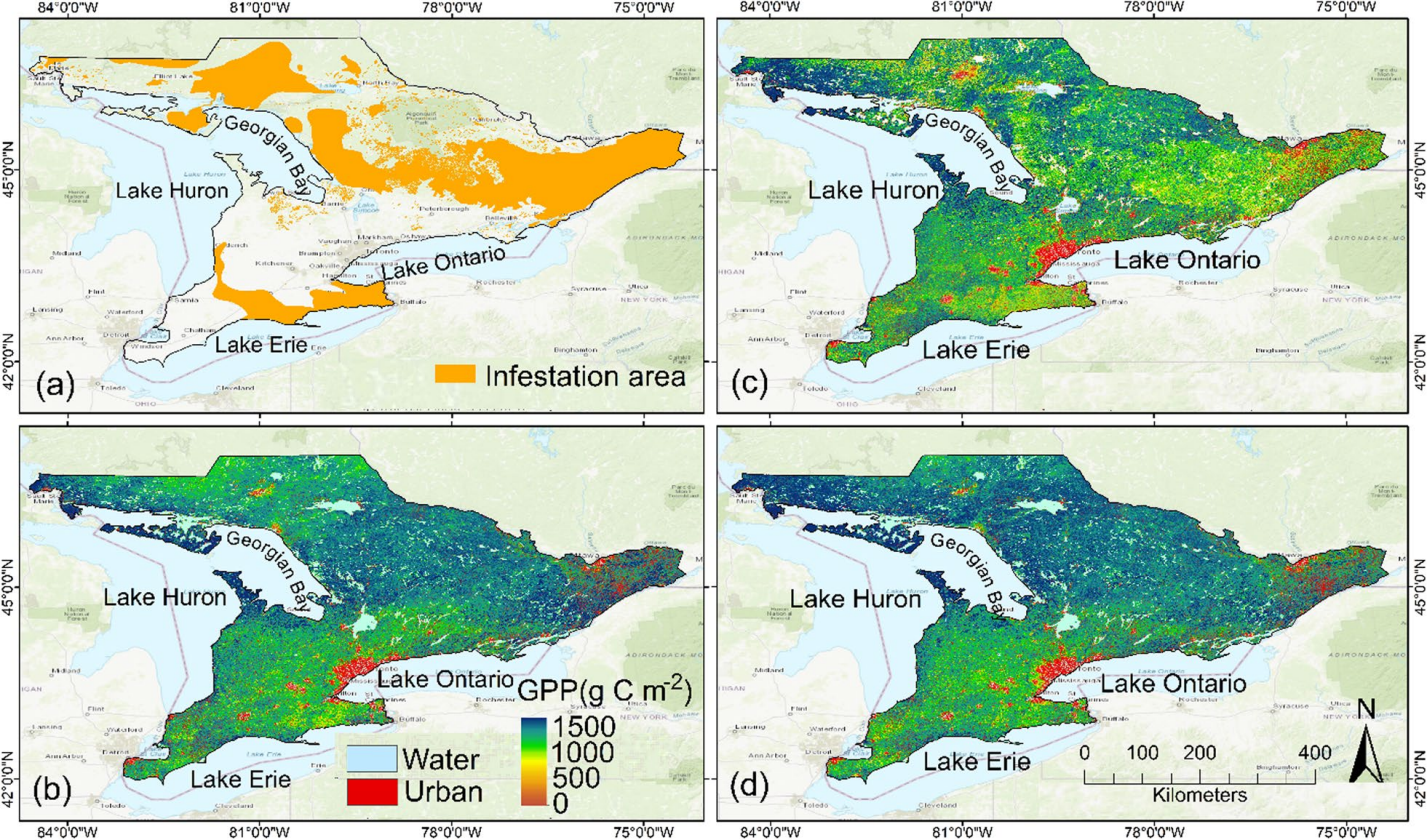
**a** The spongy moth outbreak areas in 2021. The spongy moth outbreak data were collected from the Ontario provincial database (Ontario GeoHub 2022). The LULC map was generated by machine learning-based GEE using Sentinel-2 remote sensing data from the composite images of the growing season of 2020. The spatial pattern of total gross primary productivity (GPP, g C m^−2^) over the growing season (April–October) for **b** 2020, **c** 2021 and **d** 2022

Spatial patterns of total GPP over the growing season (April–October) for 2020, 2021 and 2022 are shown in Fig. 9. These spatial patterns of GPP clearly showed the severely impacted areas and extent of decline in photosynthetic C update in the region where almost all deciduous and mixed forests were impacted. Southern areas which had a higher proportion of deciduous tree species were more severely impacted. These areas were in the north of Lake Erie and west of Lake Ontario (Fig. 9c). However, low values of GPP as shown by yellow color were prevalent almost all over the study region, except in the central and far northwestern parts that were dominated by conifer species. Overall, these results showed 35 (36)% decrease in total GPP over the growing season for deciduous forests in 2021 when compared to pre-infestation (post-infestation) years. A similar GPP decline for mixed forests was 23 (21) % in 2021 when compared to pre-infestation (post-infestation) years (Table 4).

## Discussion

Remotely sensed LAI measurements have been widely used to observe the intensity and extent of defoliation in deciduous and mixed forests (De Beurs and Townsend 2008). LAI measurements also provide direct quantification of leaf properties, photosynthetic activity, C uptake (Jarlan et al. 2008; Boussetta et al. 2013; Alton 2016; Brown et al. 2020) and often used to estimate vegetation biomass utilizing remote sensing-based models (Zolles et al. 2021). Our study results showed that the mean LAI values for deciduous forests decreased by about 25% in 2021 as compared to the pre-infestation LAI values in 2020, and by about 22% as compared to the post-infestation LAI values in 2022. It provided an indication of the severity of the impacts of spongy moth infestation on forest growth and productivity. We used these LAI values as a key indicator to observe the spatial patterns and the extent of spongy moth infestation. It helped us to observe the trajectory and dynamics of defoliation and to determine the timing and extent of canopy recovery when larvae or caterpillars were transformed into pupa and adult moths after a few weeks (Latifovic and Arain 2024). We also used these LAI values to calculate remote-sensing based GPP across the region (Sun et al. 2021). We found a strong positive correlation between LAI and remote sensing based GPP values with *R*^2^ values of 0.90, 0.76, 0.86 and 0.67 for agricultural lands, coniferous forests, deciduous forests and mixed forests respectively and significance level (*p*) values of ≤ 0.005 (data not shown). Similar strong correlations between LAI and GPP have also been found by other researchers (e.g. Qu et al. 2018; Zhang et al. 2021; Chen et al. 2023).

Our analysis showed the intense and widespread nature of the 2021 spongy moth infestation in the region where deciduous and mixed stands experienced large-scale defoliation resulting in 35% and 22% decrease in mean daily GPP values as compared to 2020 and 2022, respectively. Our study not only supported the earlier inferences that 2021 infestation was as record disturbance event in North America (Embrey et al. 2012; CFIA 2021; Chung et al. 2021; Gooderham et al. 2021; Government of Canada 2021; MNRF 2021; MNDMNRF 2022; TRCA 2022; Clark et al. 2022; Foster et al. 2022; Coleman and Liebhold 2023; Latifovic and Arain 2024), but it also provides quantitative assessment of the photosynthetic C uptake reduction across the region due to defoliation (Dymond et al. 2010; Medvigy et al. 2012; Kretchun et al. 2014). These C uptake reduction estimates have significance because in recent years most of the terrestrial C cycle studies in the literature have been reporting an increase in vegetation C uptake due to warmer temperatures, longer growing seasons and CO_2_ fertilization effects (Goodale et al. 2002; Harris et al. 2016; Birdsey et al. 2019; Fei et al. 2019; Ameray et al. 2021; Quirion et al. 2021). Our study has highlighted how C sequestration of deciduous and mixed forest ecosystems in eastern North America, specifically in the Great Lakes region, might be impacted by a major natural disturbance event. Such natural disturbance events are expected to increase in frequency and intensity in the future due to climate change (Pureswaran et al. 2018; IPCC 2021; Harvey et al. 2022; Kalamandeen et al. 2023). They will have adverse consequences for biological C sinks to offset greenhouse gases (GHG) emissions to achieve net zero C emission gaols.

Our study also showed that in the Great Lakes region, conifer forests have much greater capacity for C sequestration as compared to deciduous and mixed forests due to their longer growth period and conducive environmental conditions in the region (Payne et al. 2019; Beamesderfer et al. 2020). Sustainable management of both deciduous and conifer forests may help to conserve and futher enhanc C uptake capacity of these forests. In this regard, our study provides systematic methodology and road map to monitor and quantify the growth and C sequestration of all major vegetation ecosystems in the region, including conifer, deciduous and mixed forests as well as agricultural lands at high (10 × 10 m^2^) spatial resolution. Because most inset infestations are speciess specific and some of them occur for short periods such as spongy moth infestations, it becomes very challenging to accuratly quantify their impacts. Our utlizaion of high-resolution Sentinel-2 satellite imagery and a light use efficiency (LUE) model to estimate GPP for the whole region was a unique effort which provided a quantitative assessment of the photosynthetic C uptake loss because of the large scale nature of this infestation. It showed that 2021 infestation caused 4.84 and 2.6 t C ha^−1^ reduction of C uptake in deciduous and mixed forests, respectively. This was a substantial potential C sequestration loss, considering the mean annual GPP of 14.0 t C ha^−1^ for Canada (Gonsamo et al. 2013; Chen et al. 2020) and 12.25 t C ha^−1^ for USA (Turner et al. 2003; Tang et al. 2010). Our estimated total C uptake loss for the whole study area of 178,000 km^2^ in 2021 was 21.1 (21.4) megatons of carbon (Mt C) when compared to 2020 (2022). This C loss amounted to ~ 11.5 (11.7)% of the Canada’s national GHG emission of 182.7 Mt C eq (670 Mt CO_2_ eq) or 52.3 (52.1)% of the Province of Ontario’s GHG emissions of 41.1 Mt C eq (150.6 Mt CO_2_ eq). However, the reader is cautioned about these extrapolated results because the defoliation is tree species dependent and there may be areas which many have not been severely impacted as well as the uncertainty associated with the remote sensing derived GPP values. Our study has also highlighted the importance of future forest conservation and management practices that should account for climatic and disturbance stresses and help to enhance the sustainability and resilience of forests to these stresses.

## Conclusions

This study quantified the impact of a serve spongy moth infestation on C sequestration in deciduous and mixed forest ecosystems in the Great Lakes region in Canada. By utilizing remotely sensed LAI as a key indicator, study assessed the onset and progression of spongy moth infestation in 2021. Study results showed a substantial decline in GPP in deciduous and mixed forests in 2021 when compared to pre- and post-infestation years i.e. 2020 and 2022. Total growing season GPP values were 1338, 868, and 1367 g C m^−2^ in deciduous forests over the study area from 2020 to 2022, respectively. Corresponding mean total growing season GPP values in mixed forests were 1208, 932, and 1175 g C m^−2^ and in coniferous forests they were 1443, 1475, and 1438 g C m^−2^ in 2020, 2021 and 2022, respectively. It showed 35 (36)% reduction in mean total growing season GPP in deciduous forests in 2021 as compared to pre-infestation (post-infestation) years. Corresponding decline in mixed forests was 23 (21)% in 2021. The whole study area (178,000 km^2^) experienced the total photosynthetic C uptake loss of 21.1 (21.4) Mt C when compared to 2020 (2022). Study results also displayed that coniferous forests consistently exhibited higher GPP values, indicating their efficient C sequestration capabilities. The methods developed in our study and their application using high resolution remote sensing data will help to improve our understanding of C dynamics of forest ecosystems in response to natural disturbances. Our results also emphasize the vulnerability of deciduous and mixed forests to insect infestations and signify the need to develop proactive and adaptive forest management practices that can enhance forest resilience to climate change. They will help to quantify regional-scale C balance and develop sustainable forest management practices to contribute to net zero C emission goals through nature-based solutions to mitigate climate change.

## Data Availability

The datasets used during this study are available from the corresponding author upon request.
